# Development of Multiple Crosslinked Polymers and Its Application in Synthetic-Based Drilling Fluids

**DOI:** 10.3390/gels10020120

**Published:** 2024-02-02

**Authors:** Jun Yang, Tengfei Dong, Jingtian Yi, Guancheng Jiang

**Affiliations:** College of Petroleum Engineering, China University of Petroleum (Beijing), Beijing 102249, China

**Keywords:** multiple hydrogen bonds, crosslinked polymer, synthetic-based drilling fluids, nanoparticles, emulsion stability

## Abstract

This study addresses the performance challenges of Synthetic-Based Drilling Fluids (SBDF) in deep wells and high-temperature environments by engineering a novel multiple hydrogen-bonded crosslinked polymer, MBAH/nano-SiO_2_. Synthesized using methyl methacrylate (MMA), butyl methacrylate (BMA), acrylic acid (AA), N-hydroxyethyl acrylamide (HEAA), and nano-silica (nano-SiO_2_), the polymer improved crosslinking density, thermal properties, particle size distribution, and colloidal stability. The development of a ‘weak gel’ structure in W/O emulsions improved rheology and electrical stability (ES), with ES values reaching up to 775 V after aging at 180 °C. Moreover, the polymer’s amphiphilic structure and the synergistic effect of nano-SiO_2_ increased emulsion film thickness and strength, further augmenting stability. The high-temperature and high-pressure filtration loss of SBDF was considerably reduced to 7.6 mL, benefiting well wall stability and reservoir damage control. This study provides crucial insights into optimizing multiple hydrogen-bonded crosslinked strategies and polymers in SBDF applications.

## 1. Introduction

As energy demand for oil and gas continues to grow and conventional reservoirs are being depleted, many engineers are directing their goals and interests towards unconventional reservoirs in high-temperature and high-pressure conditions, such as shale gas and geothermal [1,2]. Drilling mud (DF) as a circulating fluid in drilling engineering usually has various roles, such as purifying the wellbore, transporting rock cuttings, balancing formation pressures, maintaining the stability of the well wall, cooling and lubricating the drill bit, and so on [3,4]. Meanwhile, traditional DFs can be divided into water-based drilling fluids (WBDF) and oil-based drilling fluids (OBDF), and WBDF is widely used in drilling conventional formations because of its advantages, such as being cheap and environmental-friendly [5,6]. However, the performance of WBDF is often greatly reduced under high-temperature conditions, so when drilling into high-temperature or shale reservoirs, engineers usually choose OBDF, which has more stable performance. However, a drawback to this approach is the poor biodegradability of OBDF using diesel and mineral oil as dispersing media, as well as the unavoidable toxicity and cost of recycling and disposal for both field engineers and the natural environment. Since the 1980s, many countries have banned diesel and toxic mineral oils globally (e.g., in the Gulf of Mexico and the North Sea), especially in environmentally sensitive areas (e.g., offshore platforms) [6]. Synthetic-based drilling fluids (SBDF) as a special OBDF have found a near-perfect equilibrium between excellent performance and biodegradability. It has been reported that SBDF typically use base oils with low aromatic content such as poly alpha olefins (PAO), synthetic paraffin (n-paraffin), linear alpha olefin (LAO), etc. These synthetic oils degrade more quickly than other oils and are, therefore, far less toxic than traditional OBDF [7]. However, high-temperature and high-pressure (HTHP) pose great challenges to the performance of SBDF, and little research has been published on SBDF additives, especially polymer applied in HTHP.

In High-Temperature High-Pressure (HTHP) oil and gas reservoirs, it is well-recognized that elevated temperatures may induce molecular chain scission and mass degradation in polymers, consequently diminishing their efficacy. Improving polymer resilience in such harsh environments, thus, constitutes a significant challenge [1,8]. Crosslinking strategies have been identified as effective means to modify the mechanical, thermal, and chemical properties of polymers. The incorporation of crosslinking groups facilitates the formation of stable, three-dimensional networks of crosslinked polymers through covalent bonding [9,10,11]. Moreover, recent studies [12,13] have highlighted that nanoparticles can introduce multiple rigid structures, significantly enhancing the physical and chemical properties of polymers. These enhancements include rheological properties, emulsion stabilization, and filtration characteristics of drilling fluids. Hence, crosslinked polymers play a pivotal role in augmenting the performance of drilling fluids, particularly in the context of HTHP oil and gas reservoirs, where they are instrumental in maintaining fluid stability and enhancing operational efficiency.

Chen et al. [14] designed and synthesized a hyper-crosslinked polymer (ACP) by amine crosslinking. Due to the formation of the hyper-crosslinked structure of the ACP, it can withstand higher temperatures in SBDF and improve the stability of water-in-oil emulsions. Lei et al. [15] N-(hydroxymethyl)acrylamide (NAM) was used as the crosslinking monomer to synthesize self-crosslinking soap-less emulsion (PMS). The self-crosslinking emulsion effectively enhanced the rheological and microporous filtration properties of the drilling fluid, resulting in a better filtration loss reduction performance at high temperatures. Li et al. [16] synthesized a novel temperature- and salt-resistant micro-crosslinked poly-ampholytic electrolyte gel. The synergistic effect of covalent micro-crosslinking enabled the DF to have excellent rheological and filtration properties even at 200 °C, which greatly improved the operational stability of the DF. Huang et al. [17] synthesized a polyamide wax (TQ-1), which formed a weakly crosslinked gel structure in OBDF by hydrogen-bonded crosslinked. It was shown that TQ-1 could increase the ES values of OBDF, greatly improve the yield point and gel strength, and greatly reduce the sedimentation factor (SF).

**Figure 1:**
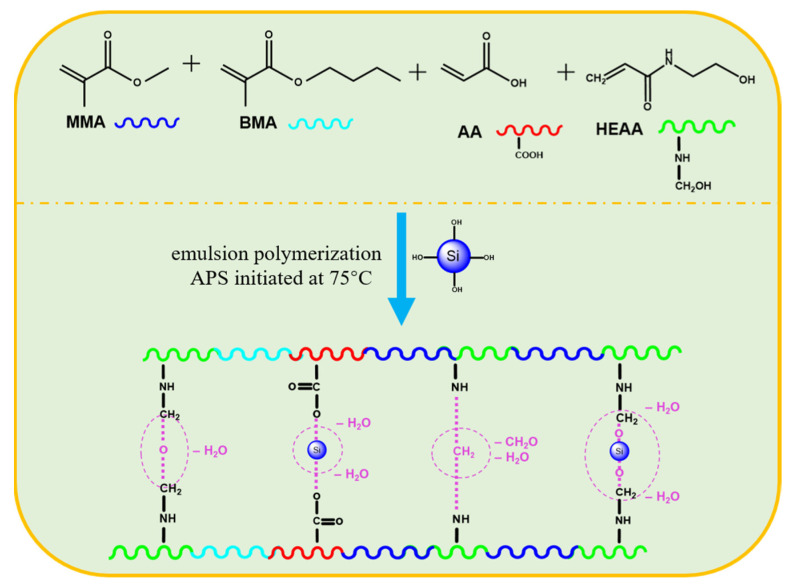
Schematic representation of the synthetic route and crosslinked mechanism of polymers (Pink curved circles indicate potential cross-linking points).

In this paper, multiple hydrogen-bonded crosslinked polymers were synthesized based on the traditional emulsion polymerization method using methyl methacrylate (MMA), butyl methacrylate (BMA), acrylic acid (AA), and N-hydroxyethyl acrylamide (HEAA) as raw materials, while nano-silica (nano-SiO_2_) was introduced (as shown in Figure 1). Fourier transform infrared spectroscopy (FTIR) was used to reveal the multiple crosslinked mechanisms and molecular structures, and the properties of the crosslinked polymers were characterized using thermogravimetric (TG), scanning electron microscopy (SEM), transmission electron microscopy (TEM), particle size distribution (PSD), and others. Finally, the performance of crosslinked polymers in SBDF was evaluated, including water-in-oil emulsion stability, drilling fluid rheology, and filtration loss reduction performance. We hope that the research will expand the application of multiple cross-linked strategies in oil and gas engineering, as well as provide referenceable implications for related researchers.

## 2. Results and Discussion

### 2.1. Characterization of Multiple Crosslinked Polymers

#### 2.1.1. Molecular Structure

**Figure 2:**
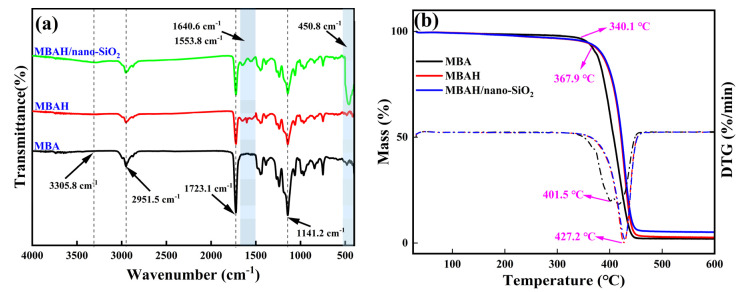
The FTIR (**a**) and TG (**b**) curves of different crosslinked polymers.

The molecular structures of the three crosslinked polymers were characterized based on FTIR, and the results are shown in Figure 2a. Among them, the stretching vibration peaks of C-H in methyl and methylene protons around 2951.5 cm^−1^, and the characteristic absorption peaks of C=O and C-O-C in the ester group at 1723.1 cm^−1^ and 1141.2 cm^−1^, respectively, confirmed that the MBAs were polyacrylate polymers. The FTIR of the MBAHs and the MBAH/ nano-SiO_2_ (the red curve and the green curves) appeared the broader peak at 3305.8 cm^−1^, which represents the complex characteristic peaks of hydroxy-OH and N-H in the amide group, and the standard amide I band (C=O) and amide II band (C-N, N-H) absorption peaks at 1640.6 cm^−1^ and 1553.8 cm^−1^, which represent the crosslinked of the HEAA to the MBA. In addition, 450.8 cm^−1^ is a typical Si-O characteristic absorption peak, indicating the successful grafting of MBAH with nano-SiO_2_. In summary, MBAH/ nano-SiO_2_ has multiple crosslinking points, such as hydroxyl and amide groups, and nano-SiO_2_ as a physical crosslinking site.

#### 2.1.2. Thermal Property

As shown in Figure 2b, the variation curves of mass loss of different crosslinked polymers with temperature, the initial decomposition temperature of MBA was 340.1 °C, the maximum decomposition temperature was 401.5 °C, and the final mass loss was 98.01%. With the introduction of HEAA and nano-SiO_2_, the initial decomposition temperature and maximum decomposition temperature increased to 367.9 °C and 427.2 °C, and the addition of crosslinked monomers and nanoparticles significantly improved the heat resistance of the polymer. In addition, the final mass loss of MBAH/ nano-SiO_2_ was 94.87%, indicating that the doping of nanoparticles enabled the polymer to maximally maintain its structural rigidity at high temperatures, which is also favorable for the potential application of the material under extreme HTHP conditions [18].

#### 2.1.3. Micro-Morphology

**Figure 3:**
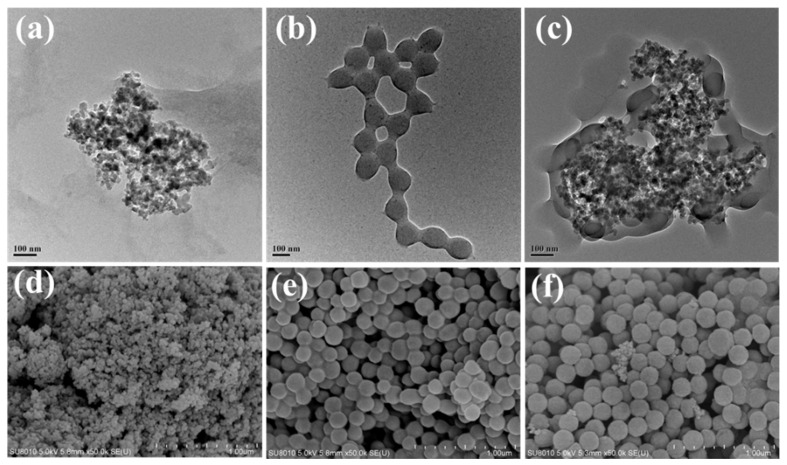
The TEM and SEM photographs of nano-SiO_2_ and different crosslinked polymers: nano-SiO_2_ (**a**,**d**); MBAH (**b**,**e**); MBAH/nano-SiO_2_ (**c**,**f**).

As shown in Figure 3, the microscopic morphology of nano-SiO_2_ and crosslinked polymer was observed by TEM and SEM. In Figure 3a,d, it was observed that the particle of nano-SiO_2_ was small, showing a typical nanoparticle aggregation state. Figure 3b,e show the crosslinked polymers without the addition of nano-SiO_2_, and it can be clearly observed that the products are largely spherical particles with irregular flexible deformation, and the particles have clear core-shell boundaries and are connected between the shells, which is due to the fact that the ester monomers are encapsulated by the hydrophilic monomers in the process of emulsion polymerization. At the same time, the hydration groups such as hydroxyl and carboxyl in the outer layer are crosslinked with each other and form a hydration network. The products after the addition of nano-SiO_2_ are shown in Figure 3e,f. It is seen that nano-sized SiO_2_ is fully doped in the polymer and forms a compact structure, and the Si-OH and -OH carried on the surface of nano-SiO_2_ can easily form physicochemical crosslinked sites with the polymer hydration shell. In summary, the introduction of chemical and physical crosslinked sites can greatly improve the particle distribution of the products, which is also favorable to the potential for plugging pores and fractures in complex formations when applied in drilling fluids [19].

#### 2.1.4. Colloidal Stability

**Figure 4:**
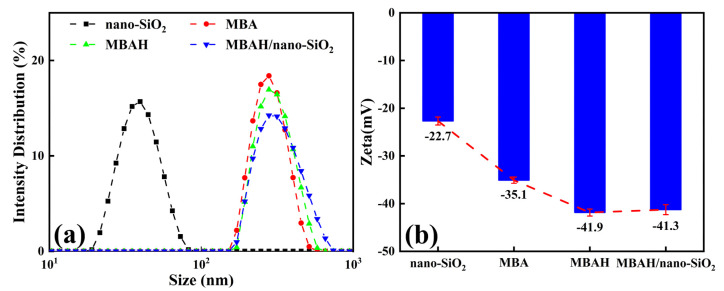
The PSD (**a**) and zeta potential (**b**) of nano-SiO_2_ and different crosslinked polymers.

The particle size distribution (PSD) and the zeta potential values can well reflect the dispersion stability of colloidal particles [20]. The PSD and zeta potential values of nano-SiO_2_ and the three crosslinked polymers are shown in Figure 4. The average particle size and zeta value of nano-SiO_2_ are about 40 nm and −22.7 mV, respectively, and the PSD is obviously monodispersed. The average particle size of MBA is 277.9 nm, and MBAH increases to about 300.2 nm because of the introduction of the crosslinked group -OH. The zeta value changed from −35.1 to −41.9 mV with the negatively charged amide group in the hydrophobic monomer. Due to the addition of nanoparticles, the average diameter of MBAH/nano-SiO_2_ increased to about 350.9 nm, and the zeta value stayed at a higher negative potential (−41.3 mV). In addition, the widening of the PSD curve can be seen in Figure 4a, representing a larger polydispersity index (PDI) of the polymers, which is attributed to the introduction of chemical and physical crosslinking sites leading to a more complex crosslinking network.

### 2.2. Performance Evaluation of Emulsions

#### 2.2.1. Shear-Thinning Behavior

**Figure 5:**
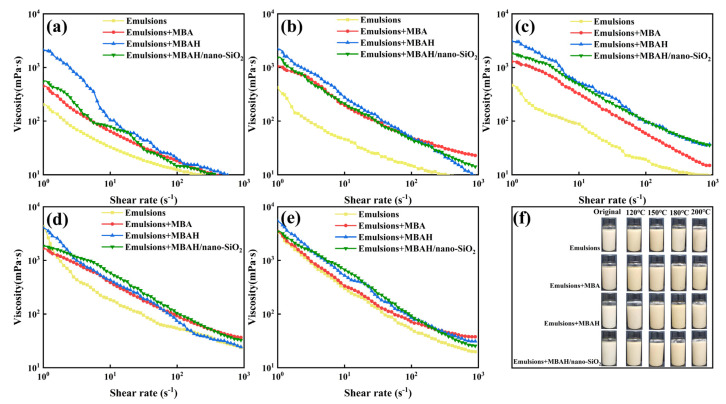
The shear-viscosity curves of synthetic-based emulsions with different crosslinking polymers added after ageing at different temperature: original (**a**); 120 °C (**b**); 150 °C (**c**); 180 °C (**d**); 200 °C (**e**); flow states (**f**).

We investigated in detail the shear rheological properties of the synthetic-based emulsions at different temperatures (original, 120 °C, 150 °C, 180 °C, and 200 °C) after the addition of 3 wt% of crosslinked polymers (MBA, MBAH, and MBAH/nano-SiO_2_), respectively. As shown in Figure 5, all emulsions exhibited typical non-Newtonian fluid behavior, and a significant viscosity decrease with increasing shear rate (shear thinning characteristics). The shear-thinning property of drilling fluids can reduce the energy loss at high shear rates, while maintaining a high viscosity at low shear to enhance the rock-chip carrying capacity [20]. Figure 5a shows the viscosity with the addition of crosslinked polymers at high shear rates tends to be the same as that of the emulsions; crosslinked polymers still increased the overall viscosity of the emulsions at low to medium shear rates. As the temperature increased to 120 °C and above 150 °C (Figure 5b,c), the viscosity enhancement effect of the crosslinked polymers in the full range of shear rates was significantly enhanced, which on the one hand was due to the high-temperature effect caused by the intensification of the Brownian motion and the disruption of the W/O structure, which resulted in the decrease of the overall stability of the base emulsions. On the other hand, it is due to the formation of micro-crosslinked structure (weak gel structure) by high temperatures [16]. The consequent increase in structural strength within the fluid somewhat inhibits the thermodynamic motion of the emulsion, so that a relatively stable viscosity reduction trend is ensured over a wide range of shear rates. However, further increase in temperature seems to break this advantage, especially above 180 °C (Figure 5d,e), where the viscosity-shear curves of emulsions tend to be close to each other, suggesting that extreme temperatures can be a major factor contributing to the change in viscosity. Under the combined pressure of extreme temperature and high shear rate, the crosslinked network structure formed by the polymer may tend to break down or disintegrate and fail to support the overall structural stability of the emulsion. In addition, all data show that the viscosity-shear curves with MBAH/nano-SiO_2_ at experimental temperatures are above the other curves, suggesting that the polymers contribute to the formation of the relatively most stable dispersion system of the emulsions, with the introduction of cross-linking groups and nanoparticles. In other words, the crosslinked polymers still improve the resistance of the emulsions to the effects of high temperatures and high mechanical forces.

#### 2.2.2. Emulsion Stability

**Figure 6:**
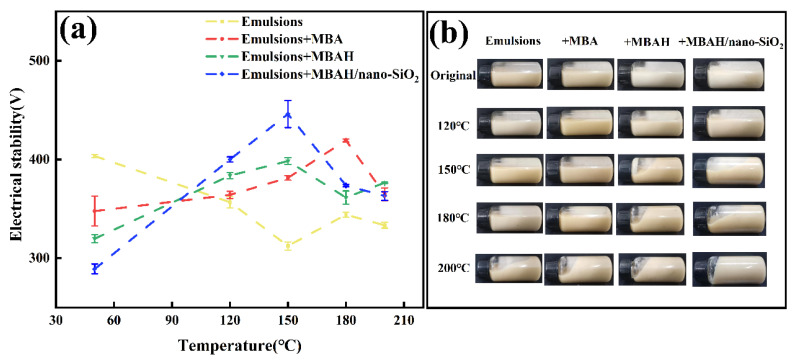
The ES values (**a**) and gel state (**b**) of base emulsions with different crosslinked polymers added after ageing at different temperatures.

The stability of the emulsion is necessary for the excellent performance of SBDF, and the ES values reflect the ability of the emulsion to resist the separation of the oil and water phases [21]. As shown in Figure 6, the ES values of the emulsions decreased with increasing temperature, from an initial 403 V at 150 °C to a minimum of 150 V, indicating that the high temperature led to the separation of the oil and water phases in the emulsions, which was extremely detrimental to the rheological stability of SBDF. The emulsions with 3 wt% crosslinked polymers showed the higher ES values (446 V) in the high temperature range (120–150 °C). This may be attributed to the amphiphilic groups of the polymers being rapidly distributed at the oil-water interface to optimize the interfacial arrangement and reduce the interfacial tension at high temperature. The crosslinking groups generated a hydrogen-bonded crosslinking network in the aqueous phase to ensure the phase equilibrium of the W/O emulsions. In addition, it has been shown [22] that the excellent interfacial effect of nano-SiO_2_ leads to its adsorption on the oil-water interfacial film to form a strong droplet “shell”, and the synergistic effect of emulsifiers increases the density and thickness of the interfacial film, so that the stability of the emulsion is greatly improved. However, the stable structure of the oil-water interface at higher temperatures (180–200 °C) seems to be dominated, but the overall ES values (up to 376 V) are still higher than that of the base emulsion, which proves that it still has a promotional effect at extreme temperatures.

### 2.3. Performance Evaluation of SBDF

#### 2.3.1. API Rheological Performance

**Figure 7:**
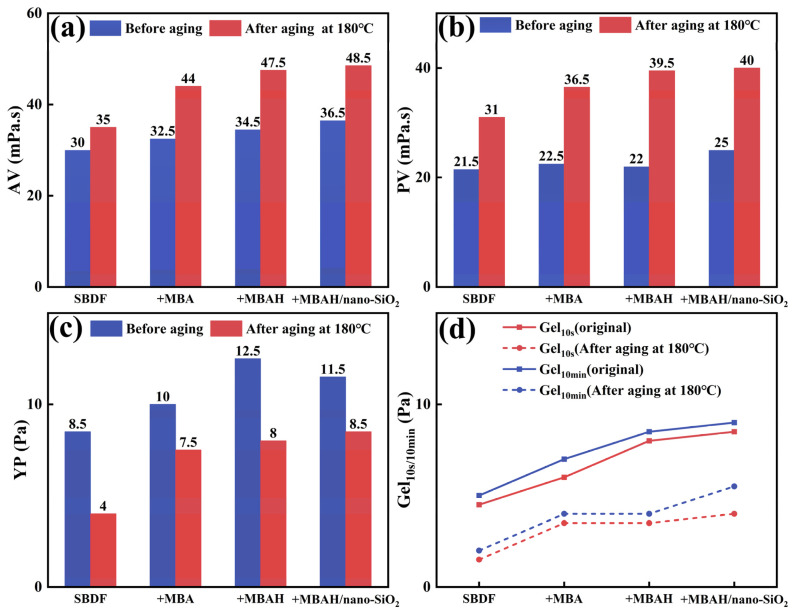
The AV (**a**), PV (**b**), YP (**c**), and Gel_10s/10min_ (**d**) before and after aging of SBDF at 180 °C.

Based on the results of the stability evaluation of synthetic-based emulsions, we investigated the effects of 3 wt% crosslinked polymers on the API rheology (including AV, PV, YP, and Gel) of SBDF (with a density of 1.2 g/cm^3^), after the 180 °C aging heat rolling for 16 h. As shown in Figure 7, with the addition of crosslinked polymer, the rheological parameters of SBDF before 180 °C were generally slightly increased, with the AV and PV increased by 6.5 and 3.5 mPa.s, respectively, the YP increased from 4 Pa to about 8.5 Pa, and the Gel_10s_, Gel_10min_ increased to 8.5 Pa and 9 Pa. The data before aging showed that the crosslinked polymers do not produce an increase in the flow resistance of SBDF, which facilitates SBDF flow into the borehole and formation. As well depth increases, formation temperature and pressure tend to destabilize the rheological properties of drilling fluids, and the YP represents the ability of the drilling fluid to suspend solid-phase particles, and maintaining the appropriate Gel facilitates the carrying of rock cuttings when circulating the drilling fluid [23]. As can be seen from Figure 7c,d, the YP after 180 °C decreased to 4 Pa, and the Gel_10s/min_ was lower than 2 Pa, indicating that the internal gel network and strength of the SBDF without the addition of crosslinking polymers were destroyed. However, the YP and Gel values of SBDF with crosslinked polymer are lower than those before aging but remain in a more suitable range (maximum YP = 8.5 Pa, Gel_10s/10min_ are 4 Pa and 5.5 Pa, respectively), which is more capable of meeting the suspending solid phase and carrying the rock cuttings in comparison. Therefore, this crosslinked polymer effectively enhances the performance of synthetic-based drilling fluids at temperatures below 180 °C.

#### 2.3.2. Filtration Performance

**Figure 8:**
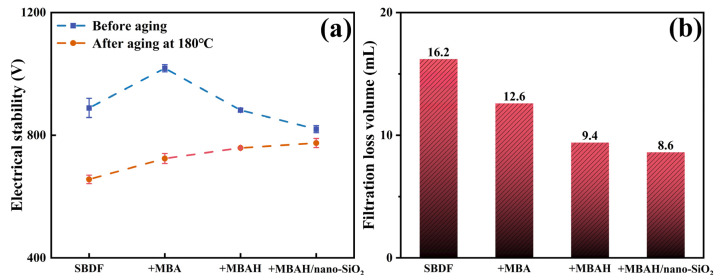
The ES values (**a**) and filtration loss (**b**) of SBDF before and after aging at 180 °C.

SBDF is often used to drill shale formations, with wellbore stability problems often occurring. The drilling fluid filtrate usually intrudes into the shale pores and the wellbore along with the natural fractures, which increases the pore pressure of the shale and leads to self-absorption phenomenon. In serious cases, the emulsifier and oil-water phase of SBDF adsorbed on the surface of shale causes emulsion plugging and wettability damage, which not only leads to the reduction of cementing quality of cement, but also influences the final production [24]. Therefore, SBDF also needs to ensure the higher emulsion ES values and lower filtrate loss volume as much as possible at HTHP. As shown in Figure 8a, the ES values of SBDF were 889 V before aging, and increased to 1018 V after the addition of MBA, while the small decrease in ES after the addition of MBAH and MBAH/nano-SiO_2_ may be caused by the introduction of hydrophilic monomers and nano-SiO_2_ in the unaged condition, which changed the oil-water ratio of the emulsion, but it is still up to more than 820 V. The emulsion stability of SBDF after aging was damaged by high temperature and decreased to about 656 V. The ES remained more stable (775 V) because MBAH/nano-SiO_2_ formed a crosslinked network in the emulsion after aging and thus effectively controlled the oil-water phase stability. In addition, the HTHP (180 °C, 3.5 MPa) of polymer-added SBDF after aging decreased linearly from 16.2 mL in the base system to 8.6 mL (Figure 8b). That is because the polymer structure contains many hydrogen-bonded crosslinked networks and rigid particles, which improves the capacities of resist oil-water segregation and structural destabilization at elevated temperatures. On the other hand, because the polymer is a deformable flexible particle and nanoparticle doped aggregate, the particle deformation and PSD distribution are wider under differential pressure, compared with the formation of a denser filter cake after deposition, which prevents the further intrusion of solid-phase particles and liquid-phase, which is conducive to the stabilization of the well wall and the control of reservoir damage [25,26].

## 3. Conclusions

This study’s synthesis of the MBAH/nano-SiO_2_ polymer, identified by FTIR spectroscopy, comprises ester, amide, hydroxyl functional groups, and nano-silica, yielding an amphiphilic structure with extensive crosslinking. Thermogravimetric (TGA) and Particle Size Distribution (PSD) analyses reveal improved thermal stability, with decomposition temperatures increasing to 367.9 °C and 427.2 °C, and particle size increasing to about 350.9 nm, suggesting enhanced HTHP sealing efficacy. Evaluations of W/O emulsions show that MBAH/nano-SiO_2_ improves electrical stability (up to 775 V post 180 °C aging) and reduces filtration loss by 50% (to 7.6 mL). The polymer forms a robust network, resilient to high-temperature damage and efficient in minimizing solid particle intrusion, promising for well wall stabilization and reservoir damage control. In our future work, we would like to work on finding even better crosslinked monomers that can be used to meet the extreme environmental conditions of formations above 180 °C.

## 4. Materials and Methods

### 4.1. Materials

The monomers used for product synthesis in this paper include methyl methacrylate (MMA), butyl methacrylate (BMA), acrylic acid (AA), N-hydroxyethyl acrylamide (HEAA), and nano-silica (nano-SiO_2_), all purchased from Aladdin Industrial Corporation (Shanghai, China). Additionally, sodium dodecyl sulfate (SDS), octylphenol polyoxyethylene ether (OP-10), and ammonium persulfate (APS) were all procured from Shanghai Energy Chemical Co., Ltd. (Shanghai, China).

The main agents used for evaluating the performance of emulsion and synthetic-based drilling fluid include natural gas synthetic oil provided by the Zhanjiang Branch of China National Offshore Oil Corporation (Zhanjiang, China). The primary emulsifier, secondary emulsifier, organophilic clay, calcium carbonate, and barite were provided by Beijing Shida Bo-Cheng Technology Co., Ltd. (Beijing, China). Additionally, sodium hydroxide, calcium oxide, and calcium chloride were of industrial grade and purchased from Shanghai Energy Chemical Co., Ltd. All reagents used in the experiments were used as received without further purification, and deionized water was produced for the experiments.

### 4.2. Synthesis of Crosslinked Polymers

The synthesis of crosslinked polymers was carried out based on the emulsion polymerization method. Initially, a mixture of SDS and OP-10 at a mass ratio of 1:2 was added to 100 mL of deionized water and mixed uniformly, followed by the addition of 1 g nano-SiO_2_ dispersed via ultrasonication for 10 min. Subsequently, MMA, BMA, AA, and HEAA (at a mass ratio of 40:20:1:3) were added sequentially to form a stable and uniform emulsion through mechanical emulsification. The mixture was then transferred to a three-necked flask and placed in a water bath heating apparatus. Nitrogen was introduced, and the temperature was raised to 50 °C, at which point APS was slowly added to initiate the reaction. The temperature was further increased to 75 °C, and the reaction was maintained for 6 h. The final product is crushed using vacuum freeze drying and was denoted as MBAH/nano-SiO_2_. Additionally, to study the crosslinking reaction mechanism, comparative polymer synthesis was conducted using the above steps, without the addition of nano-SiO_2_, denoted as MBAH, and without both HEAA and nano-SiO_2_, denoted as MBA.

### 4.3. Characterization of Crosslinked Polymers

The three polymer samples (MBA, MBAH, and MBAH/nano-SiO_2_) from Section 4.2 were dried at 80 °C. Their molecular structures were then acquired using a Magna-IR 560 spectrometer (Nicolet, Madison, WI, USA) in the wavelength range of 4000 cm^−1^ to 400 cm^−1^. Additionally, thermal properties of the samples were analyzed using a TG/DSC thermal analyzer (Netzsch, Selb, Germany).

The same three polymer samples (MBA, MBAH, and MBAH/nano-SiO_2_) were diluted in deionized water to a final concentration of 0.1%. Subsequently, their microstructures were examined using a transmission electron microscope (TEM) (FEI, Hillsboro, OR, USA) and a scanning electron microscope (SEM) (Hitachi SU8010, Hitachi, Tokyo, Japan). Furthermore, particle size distribution and zeta potential of the liquid samples were measured using a Nano ZS particle size analyzer (Malvern Instruments Ltd., Malvern, UK).

### 4.4. Performance Evaluation Methods for SBDF

#### 4.4.1. Preparation of Emulsions and SBDF

Based on the formulations in Table 1, synthetic-based emulsions and SBDF were prepared, followed by experiments to evaluate the rheological properties, electrical stability, API rheology, and fluid loss characteristics of the emulsions and SBDF. To simulate high-temperature reservoir formation conditions, the prepared synthetic-based emulsions and SBDF were sealed in stainless steel aging cells and subjected to 16 h of rolling heating using a GW300-X high-temperature rolling oven (Qingdao Tongchun Instrument Company, Qingdao, China), with the heating temperature set according to requirements.

#### 4.4.2. Emulsion Rheology Testing

The rheological properties of the emulsions were investigated using a HAAKE MARS60 rheometer (Thermo Fisher Scientific, Waltham, MA, USA) equipped with a C35 1°/Ti rotor. The gap between the parallel plates was 0.053 mm and the test temperature was 25 °C. Stepwise rotational measurements were performed by varying the shear rate from 1000 to 1 s^−1^ to evaluate the viscosity and shear thinning behavior.

#### 4.4.3. Electrical Stability Testing

The electrical stability (ES) values of the emulsions were measured using a DWY-2 electrical stability tester (Qingdao Tongchun Petroleum Instruments, Qingdao, China) with a peak current of 61 µA. Each emulsion sample was measured at least three times, and the mean with standard deviation was recorded. The ES value represents the voltage at which the emulsion is disrupted by the peak current. In general, the higher the ES value, the more stable the emulsion. The American Petroleum Institute (API) recommends a minimum electrical stability value of 200 V for synthetic drilling fluids.

#### 4.4.4. API Rheological Testing

The rheological parameters of the synthetic-based drilling fluids were determined according to the API RP 13B-2-2014 operational standard [27]. The rheological parameters of the drilling mud before and after hot rolling aging were measured using a ZNN-D6 six-speed rotational viscometer, including apparent viscosity (AV), plastic viscosity (PV), yield point (YP), and gel strength (Gel10s, Gel10min).

The formulas for calculating the rheological parameters are as follows:

(1) $$\mathrm{AV}=0.5\theta_{600}\;(mPa\cdot s)$$

(2) $$\mathrm{PV}=\theta_{600}-\theta_{300}(mPa\cdot s)$$

(3) $$\mathrm{YP}=0.5\;(\theta_{300}-P)(Pa)$$

where θ_600_ and θ_300_ are the readings at 600 rpm and 300 rpm, respectively.

Additionally, Gel_10s_ and Gel_10min_ are the 3 rpm readings after being static for 10 s and 10 min, respectively.

#### 4.4.5. Evaluation of Filtration Performance

Using the API RP 13B-2-2014 operating standard [27] with a GGS71-A High-temperature and High-pressure Filtration Loss Apparatus (Qingdao Tongchun Instrument Co., Ltd., Qingdao, China), the aged SBDF was rapidly stirred and dispersed for 10 min. It was then loaded into the HTHP filtration loss apparatus for heating to the required temperature. Once the temperature stabilized, a pressure differential of 3.5 MPa was applied to deposit SBDF on the filter paper to form a filter cake, while simultaneously recording the volume of filtrate from the SBDF over 30 min (a relative measurement of static invasion into the porous formation of the wellbore).

## Figures and Tables

**Table 1:** Formulation of emulsions and SBDF.

Component	Emulsions	SBDF
Natural gas synthetic oil	280 mL	280 mL
Primary emulsifier	14 g	14 g
Secondary emulsifier	10.5 g	10.5 g
Calcium chloride solution (25 wt%)	70 mL	70 mL
Organophilic clay	7 g	10.5 g
Calcium carbonate	/	7 g
Calcium carbonate	/	17.5 g
Barite	/	200 g

## Data Availability

Data is contained within the article.
